# YouTube as a Source of Patient Information for Cervical Spine Fractures: A Content Quality and Audience Engagement Analysis

**DOI:** 10.3390/healthcare12242492

**Published:** 2024-12-10

**Authors:** Michał Krakowiak, Kaja Piwowska, Justyna Fercho, Rami Yuser, Maks Jagodziński, Klaudia Kokot, Andrzej Bilyk, Aleksandra Maj, Jarosław Dzierżanowski, Jacek Nacewicz, Piotr Zieliński

**Affiliations:** 1 Neurosurgery Department, Medical University of Gdansk, 80-952 Gdansk, Poland; michalkrakowiak@gumed.edu.pl (M.K.); olasalzburg@gmail.com (A.M.); jaroslaw.dzierzanowski@gumed.edu.pl (J.D.); pziel@gumed.edu.pl (P.Z.); 2 Student’s Scientific Circle of Neurosurgery, Neurosurgery Department, Medical University of Gdansk, 80-952 Gdansk, Poland; kaja.piwowska@gumed.edu.pl (K.P.); m.jagodzinski@gumed.edu.pl (M.J.); klaudia.kokot@gumed.edu.pl (K.K.); andrii.bilyk@gumed.edu.pl (A.B.); 3 Emergency Department, Medical University of Gdansk, 80-952 Gdansk, Poland; justyna.fercho@gumed.edu.pl; 4 Neurosurgery Department, Wojewódzki Szpital Specjalistyczny, 76-200 Słupsk, Poland; jnacewicz@poczta.onet.pl

**Keywords:** YouTube, cervical, spine, fracture

## Abstract

This study aimed to assess the quality of YouTube (YT) videos providing medical information on cervical spine fractures; secondly, a comparison of two timeframes has been conducted. Using Google Chrome with privacy settings to minimize personalization, two searches were conducted on 20 July 2021 and the second one on 10 April 2024 using various terms related to cervical spine injuries. Videos were evaluated using the DISCERN (Quality Criteria for Consumer Health Information), GQS (Global Quality Score), and JAMA scoring systems. In total, 91 videos were included. Mechanisms of injury were the most frequent video content (n = 66), and postoperative pain occurred the least (n = 6). The mean DISCERN score of 43.26 (std = 11.25), mean GQS of 2.67 (std = 0.74), and mean JAMA score was 2.2 (std = 0.68). Inclusion of treatment options had an odd ratio of 21.72 for a better-quality video. The largest number of videos was provided by physicians (n = 24). In DISCERN, risks of treatment were graded lowest = 1.9. Newer videos achieved higher scores in the DISCERN, GQS, and JAMA scoring systems reaching 52.5, 3, and 2.75, respectively. These scores suggest inadequate information provision in the videos, hindering patients’ understanding of their condition. Due to insufficient information presented in current videos, patients are not fully informed.

## 1. Introduction

In present times, a plurality of sources of information can be more confusing and problematic than the actual lack of information when it comes to resolving dilemmas or expanding knowledge. Everyday users of mass media are forced to verify the credibility of widely accessible information [1]. The problem is not only caused by everyday people commenting on social media or forums but also by editors of websites who spread fake news.

One of the most popular websites used as a source of information is YouTube (YT), which is a platform existing since 14 February 2005 offering a broad spectrum of knowledge in the form of videos from many fields of science including medicine. YouTube is a source of many useful, substantive, and well-developed issues. For instance, Mamlin and colleagues [2] forecasted that social media platforms like YouTube would see extensive utilization for exchanging healthcare information between providers and consumers, enabling peer-to-peer patient support, and bolstering public health surveillance efforts. However, we cannot expect the average viewer to be endowed with a suitable level of knowledge to find specific valuable videos and distinguish them from doubtful content. In the age of the internet, it is essential to evaluate virtual sources of information in terms of the quality and essence that these sources convey. Studies assessing the quality of YT videos have been conducted for several subjects that are within the scope of orthopedic surgeons and neurosurgeons, for example, Bankart lesion [3], posterior cruciate ligament [4], neck pain [5], and carpal tunnel [6]. But there are no articles assessing the quality of YouTube videos about cervical fractures (CFs). And there is a lack of appropriate information about cervical spine fractures on YouTube. This is essential because, despite the low incidence rate, cervical spine injuries are very serious medical conditions and can often lead to death or tetraplegia, with ASIA-A (American Spinal Injury Association) patients representing up to 43% of incidences and ASIA-B up to 37% [7]. Spinal cord injury (SCI) has a dramatic influence on virtually every aspect of the life of the person who suffers from it. The costs of therapy are high, and treatment is long-lasting; frequently, surgery is the only option patients have. Spine injury, by its definition, is a temporary or permanent change in its function, which occurs due to an external force. Different types of damage can be distinguished due to the direction of the force: Flexion, Extension, Rotation, Lateral bending, Distraction, and compression. Often, the different types overlap in the case of a complex force, for example, during car accidents. Injuries to the C5 vertebrae and higher can be fatal because damage to the respiratory center can occur, leading to respiratory failure and often death. The most common etiologies are car accidents and falls from heights. For non-traumatic causes, the most common cervical cord injury is compression fractures in people with osteoporosis or bone tumors [8]. Cervical spinal cord injuries are extremely dangerous and often fatal in their consequences, so proper prevention in the spread of fake news is extremely important. Therefore, it is crucial to examine the quality of information available on YouTube, a platform primarily used by non-medical individuals. Most of the users who are watching medical videos on YouTube are making decisions related to whether to consult a doctor or not [9]. The lack of accurate and reliable information on such an important subject poses a significant threat. It may lead to improper behavior in critical situations such as accidents. This, in turn, can result in the deterioration of the condition of the person involved in the accident and even death, which could be easily prevented if patients were properly educated and had access to reliable sources of information. For the reasons outlined above, the objective of this study is to evaluate the quality of YouTube (YT) videos providing medical information on cervical spine fractures. Furthermore, this study seeks to compare data across two distinct timeframes to assess whether the quality has improved over time.

## 2. Materials and Methods

### 2.1. Search Strategy and Data Collection

A YouTube search was conducted on 20 July 2021 using Google Chrome browser in “Incognito mode”, which is a feature that allows users to watch videos without their viewing activity being saved to their account history. Cleared search history, no Google account attachment, and “Relevance-Based Ranking” were used to avoid the personalization of results. “Relevance-Based Ranking” on YouTube refers to the algorithm that determines the order in which videos are displayed to users in search results, recommendations, and other parts of the platform. A comparative second search was conducted on 10 April 2024 to include any new videos that had been uploaded and verify the quality difference in the time period. The time frame was based on the rapid increase in YT publications on PubMed from 2020 reaching 468, 615 in 2021, and 672 in 2022, which increased yearly up to 707 in 2023 (https://pubmed.ncbi.nlm.nih.gov/?term=Youtube+ (accessed on 28 October 2024)). The following search terms were used for data extraction: “cervical spine fracture”, “cervical spine injury”, “broken cervical spine”, “broken neck”, “neck trauma”, and “c spine trauma”. All researched data were publicly available, and this study did not include human nor animal participation, and thus no ethics committee nor YouTube permission was necessary. For the reasons outlined above, the objective of this study is to evaluate the quality of YouTube (YT) videos providing medical information on cervical spine fractures. Furthermore, this study seeks to compare data across two distinct timeframes to assess whether the quality has improved over time.

### 2.2. Inclusion and Exclusion Criteria

Inclusion criteria: All videos searched by using the above-mentioned terms were taken under consideration. Pranks, news reports, and duplicates were removed; videos in parts were treated as one production (views, time, and comments added together). Non-English videos were excluded, and videos with subtitles provided by the authors (non-automatically generated) were included.

### 2.3. Variables Extracted

The source of upload (physician, hospital/clinic channel, health channel, patient, other), video duration, and substantive content of every video were analyzed. The comparable protocol of video content was extracted (symptoms, treatment, animations, diagrams, etc.) according to previous studies [10,11,12,13,14,15,16,17,18,19,20,21]. In addition, characteristic features relevant for the medical and non-medical viewer (mortality, post-injury rehabilitation, neuropathic pain, steroid use, spinal cord injury, spinal shock) were chosen and evaluated based on raters’ medical experience. To investigate the quantitative information, the “VidIQ Vision for YouTube” plug-in was used. Audience engagement content (likes, dislikes, views, and channel subscriptions) was extracted.

### 2.4. Scoring System

The team of four raters independently evaluated the videos using the DISCERN (Quality Criteria for Consumer Health Information), GQS (Global Quality Score), and JAMA (the Journal of the American Medical Association) scoring systems [22,23,24]. Consistency was evaluated with the Intraclass Correlation Coefficient (ICC), with the use of a two-way random effects model for k multiple raters for DISCERN and GQS and JAMA.

The DISCERN criteria is a validated instrument to evaluate the reliability of health information. It consists of 16 questions which indicate a crucial characteristic of high-quality medical publications. In each of the indicators, a video can score from 1 to 5 points.

Weil et al. omitted the final question in DISCERN and interpreted the result based on the total number of points, where the minimum score is 15 and the maximum score is 75 [25].

A score between 63 and 75 points indicates an excellent rating, 51 and 62 points indicates a good rating, 39 and 50 points indicates an average rating, 27 and 38 points indicates a poor rating, and 16 and 26 points indicates a very poor rating [26]. “Excellent” and “good” quality videos are considered to be a useful source of information and helpful for the patient to maintain high-quality data on the illness and treatment options; the “average” video group is considered to be useful, but a patient would need an additional source of information; and the groups that are “poor” and “very poor” are not considered to be useful and should be avoided by the patient. The JAMA score ranges from 0 to 4 points judging the publication for the following features: authorship (authors, contributors, affiliations, and credentials), attribution (references and sources used for the content and copyright information), disclosures (ownership, sponsorship, advertising, commercial funding, and potential conflicts of interests), and currency (dates of posted and updated information). The GQS is a 5-point scale evaluating the quality and flow of the video. A score of 1 is classified as “poor quality, not at all useful for patients”; 2 as “generally poor quality, very limited use to patients”; 3 as “moderate quality, somewhat useful to patients”; 4 as “good quality, useful for patients”; and 5 as “excellent quality, highly useful to patients”.

### 2.5. Audience Engagement

We evaluated videos in the aspects of popularity, using the Video Power Index (VPI) according to the following formula introduced by Erdem et al. [10]:

VPI = (likes*100/(likes + dislikes))*(views/day)/100(1)

### 2.6. Statistical Analysis

For statistical analysis, PQStat v.1.8.0 (PQStat Software, Poznań, Poland) was used [27]. Verification of the data was conducted using the Shapiro–Wilk test and then analyzed according to the test results. Descriptive statistics included arithmetic mean, median, range, and standard deviation (std). Other tests included the following: Spearman’s rank correlation coefficient, Fisher’s exact test, and the Mann–Whitney U (UMW) test where appropriate. The DISCERN score was subjected to a multivariate analysis (logistic regression) against the qualitative aspects included in the video. Variables that were significant in univariate statistics (determined by the UMW test) were included in this analysis. Microsoft 365 (Microsoft Corporation ver.18.2008, Redmond, WA, USA) was used to create graphical illustrations. The Intraclass Correlation Coefficient (ICC) was selected to compare reliability between raters. Based on the dataset, we selected and calculated the two-way random effects model for multiple raters, according to the guidelines presented by Koo [28].

## 3. Results

After the application of the inclusion and exclusion criteria, 65 YT positions were included after the first search and 26 more videos during the second evaluation giving a total of 91 videos. Overall, video providers were divided into five groups: health channel n = 40, physician n = 24, hospital/clinic channel n = 17, non-medical (other) n = 6, and patient n = 4.

Videos produced in the USA constituted 60% of videos (n = 55), with the UK and India providing 8% (n = 7) of videos each. The Netherlands and South Africa had n = 2 each. There was one video from Canada, Switzerland, Thailand, Italy, Denmark, Egypt, and Germany. The country origin of 11 videos was untraceable. Descriptive statistics of all videos are presented in Table 1.

The ICC for consistency was calculated for all rating systems (*p* < 0.01). For the DISCERN ICC (2, k), it was 0.93 (CI—95% + 95%: 0.91–0.95). For the GQS ICC (2, k), with k for the number of raters, it was 0.84 (CI—95% + 95%: 0.77–0.89). For the JAMA ICC (2, k), it was 0.76 (CI—95% + 95%: 0.66–0.83). Spearman’s rank correlation coefficient was calculated between all three rating systems (*p* < 0.01):

Mean DISCERN/JAMA: r = 0.84 (CI—95% + 95%: 0.76–0.9);

Mean DISCERN/GQS: r = 0.81 (CI—95% + 95%: 0.72–0.88);

Mean GQS/JAMA: r = 0.74 (CI—95% + 95%: 0.62–0.82).

All three scales were analyzed with the above video descriptive statistics using Spearman’s rank correlation coefficient. All three scales had a negative linear correlation with the time of upload. DISCERN and JAMA had a negative linear correlation with the number of views, comments, and likes. Data are presented in Table 2 and Table 3.

All three scales had a positive linear correlation with the duration of videos: DISCERN, r = 0.5 (CI: 0.3–0.6); the GQS, r = 0.39 (CI: 0.19–0.56); and the JAMA, r = 0.45 (CI: 0.3–0.6).

The mean DISCERN score of the four raters was 43.26 with std = 11.25. The mean GQS was 2.67 (std = 0.74). The mean JAMA was 2.2 (std = 0.68). Most videos were poor, with n = 35. Moreover, 26 videos were average, 23 good, 4 very poor, and 3 were graded excellent.

**Figure 1:**
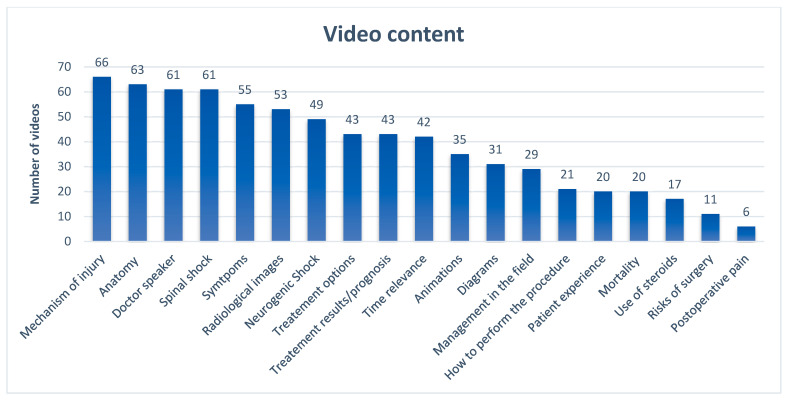
Video content for cervical spine trauma.

The overall mean DISCERN for a single question (Q) was 2.7. The highest rated DISCERN Qs were Q1 and Q2 (“are the aims clear?” and “does it achieve its aims?”) achieving 4.12 and 4.06, respectively. The lowest grade was Q11 = 1.9 (risks of treatment). The content mentioned in videos considering cervical spine fractures is presented in Figure 1.

Treatment options, treatment results, diagrams, and the physician speaker were statistically relevant in UMW tests in all scales. A multivariate analysis (logistic regression) model was built dividing videos based on the DISCERN score (>42 was deemed high and ≤42 was considered low) based on a previous evaluation method [18]. The results are presented in Table 4.

In order to compare the search results from 2021 and those found in 2024, a UMW test for DISCERN/GQS/JAMA was performed. Results were statistically significant in all three scoring systems, showing newer videos to be higher quality (Table 5).

**Figure 2:**
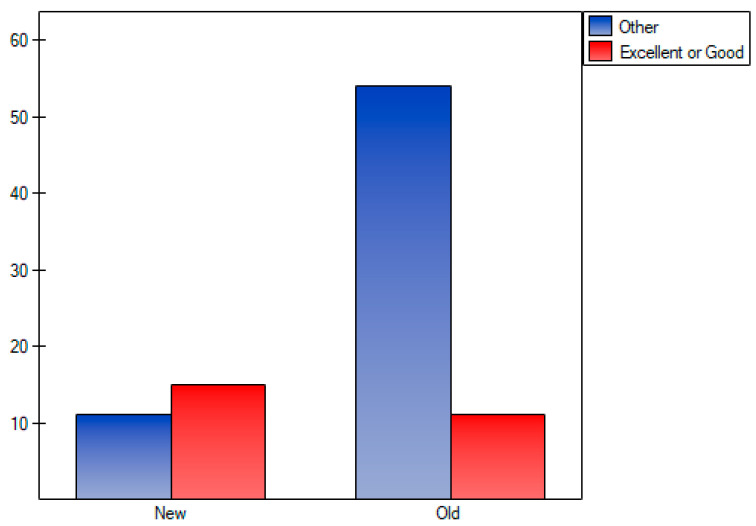
Quality of video based on DISCERN grading.

Video provider differences were statistically insignificant between groups. Videos were grouped based on their DISCERN grading into two groups: excellent or good, other, and evaluated with the use of Fisher’s exact test. Results were statistically significant *p* < 0.01 (Figure 2).

When comparing the content in Fisher’s exact test of the two video groups, statistical differences occurred as follows: mechanism of injury information increased from 66.15% up to 88.46% of videos. Symptoms increased from 52.31%to 80.77%, radiological images from 47.69% to 84.62%, and the diagram occurrence from 26.15% to 53.85%. Information on conservative treatment increased from 35.36% to 73.08% and mortality from 15.36% to 38.46%. Fewer animations (46.15% vs. 19.23%) were seen in newer productions. The occurrence of spinal shock decreased from 61.54% to 34.62%. In the UMW test, the newer videos were longer (median 2407.5 s vs. 406 s).

## 4. Discussion

Fractures of the cervical spine are uncommon and account for only 3% of trauma patients [29]. Still, 3-month mortality after CFs ranges from 2% to 18% depending on the patient’s age. The peak incidence is between the second and third decades of life with males being more frequently affected. The major cause of injury are motor vehicle accidents, constituting more than 60% of cases [30]. The incidence of CFs has increased over the years, creating a higher financial burden even if the length of stay has decreased [31]. In total, 91 videos matched the criteria, which is more than in the work considering intracranial aneurysms or hydrocephalus [32,33]. Neurosurgical topics still do not seem to be as eagerly evaluated as topics such as vaccination and immunization or organ donation for which over 300 videos were analyzed [34]. Surgical treatment in cervical trauma decreases mortality in selected cases [35]. Patients or their families might search for information about cervical spine trauma on the internet to better understand their medical condition. YouTube videos, as one of the easier sources for acquiring information, have been evaluated for proper knowledge using the DISCERN, GQS, and JAMA score. The mean DISCERN score for CF videos, based on the ratings of four raters, was fair. Still, only 3% of videos were excellent. Hydrocephalus videos had only one excellent video, and in abdominal aneurysms, the majority of analyzed videos were graded poor (41%), and none were exceptional [11]. Even with topics more broadly affecting society such as insulin resistance, 50% of videos were poor and only 2% were excellent [36]. In educational videos for insulin pen injections posted on YouTube, 55.1% of them contained useful information and 44.8% of them were considered misleading [37]. The JAMA scoring system is based on four core fundamentals such as authorship, attribution, disclosure, and currency to reasonably judge whether the material is credible or reasonable. Alone, the JAMA score was higher in comparison to that for rotator cuff repair and carbon monoxide poisoning but lower than that for vascular YT video topics [14,16,20,33].

The most commonly mentioned topics were mechanisms of injury and anatomy, while the least covered topics included postoperative pain, risk of surgery, and mortality. This might suggest that there is insufficient information about the negative aspects of treatment. The absence of sufficient facts regarding treatment risks and potential complications is a concern highlighted in numerous papers analyzing medical YouTube videos accounting for up to 10% of assessed videos about stroke as well as 13% of videos about meningioma and 20% videos about AVM [18,19,20]. If present, mostly they are described poorly [21]. Moreover, only 18.7% of YouTubers mentioned the controversial topic of steroids. Although recommendations to use methylprednisolone are based on a weak strength of evidence [38], the implementation of this topic should be mentioned when discussing the topic of cervical fractures, where spinal cord injury is of high risk.

The timing of surgery as an essential part of treatment and occurred in 46.2% of the evaluated material. Although early decompressive surgery (≤24 h after injury) is strongly recommended, there are still discrepancies in the definition of ultra-early decompression time. Yet evidence suggests that faster intervention improves the total ASIA motor score compared with late surgery [39]. CF videos’ mean time was much longer in comparison to that of disc herniation videos that lasted for 6.59 min [13], rotator cuff repair videos with a mean of 7.7 min [14], narcolepsy videos lasting for 7.75 min [15], or carbon monoxide poisoning videos lasting for 5.83 min [16]. On the other hand, there are longer videos like abdominal aortic aneurysm ones that have a mean length of 23.24 min [11].

With time, the quality of CF videos increased, showing that materials can be improved. In contrast, materials on abdominal aneurysms were still rated with a mean of GQS 3 at a later evaluation [40]. A search in 2023 once again examined the quality of lumbar disc herniations where the authors concluded that the overall quality is low and there is limited diagnostic and treatment utility [41]. Three years after, Szmuda et al. evaluated stroke videos in 2020, and a study on thrombectomy for stroke treatment was conducted with comparable results with the mean DISCERN score reaching 41.43 [18,42].

The limitations of this article are the unequal amount of videos in the two conducted searches and how only 91 videos were taken into consideration in total. Secondly, the authors are aware of the fact that not all audience members participate in liking/disliking and that there is a high possibility of one person re-watching the same video multiple times. These limitations might lower the number of included videos but are necessary to be applied. Moreover, duplicate videos were eliminated, and videos split into parts were considered as a single production, with their views, duration, and comments combined.

## 5. Conclusions

Due to insufficient information presented in current videos, patients are not fully informed and thus cannot fully understand the nature of their condition and its health implications relying only on their knowledge from YouTube. Of course, we are fully aware that patients do not fully rely on information presented on one portal; however, the omission of clinically important information is a serious issue. Although the quality has increased, future efforts should prioritize creating high-quality videos. Materials should be either presented by medical specialists or made with consultation of one, reflecting current knowledge and EBM including both the positive and negative aspects of surgical intervention. Potential benefits for authors/creators of videos might be due to the usage of AI instruments. To some degree, this might help to receive more accurate medical information and improve the quality of YouTube videos. Patients and/or their family members should be well informed to ensure conscious decisions in further therapy.

## Figures and Tables

**Table 1:** Descriptive video statistics.

	Mean	Median	Std	Min	Max
**Views**	30,442.9	3125	100,530.6	8	853,343
**Likes**	212.6	27	665.1	0	5480
**Dislikes**	9.5	1	36	0	312
**Comments**	26.8	2	81	0	511
**Subscribers**	475,323	19,600	2,932,976	61	27,100,000
**Time (s)**	1449	559	2843	42	24,042
**Days since upload**	1227	1103	867	13	3366
**VPI**	25.3	4.2	75.1	0	603.8

**Table 2:** Spearman’s rank correlation coefficient between scoring systems and time since upload.

	DISCERN/Upload Time	GQS/Upload Time	JAMA/Upload Time
r	−0.42	−0.21	−0.34
*p* value	<0.01	0.04	<0.01

**Table 3:** Spearman’s rank correlation coefficient correlations.

	DISCERN/Views	JAMA/Views	DISCERN/Comments	JAMA/Comments	DISCERN/Likes	JAMA/Likes
r	−0.36	−0.33	−0.26	−0.27	−0.27	−0.22
*p* value	<0.01	<0.01	0.01	0.01	0.02	0.034

**Table 4:** Multivariate analysis results of video content.

	Coefficient B	−95% CI	+95% CI	Wald Test	*p* Value	Odds Ratio	−95% CI	+95% CI
Treatment options	3.08	1.67	4.48	18.41	<0.01	21.72	5.32	88.66
Treatment results	0.86	−0.626	2.34	1.28	0.26	2.36	0.53	10.42
Diagrams	1.8	0.29	3.31	5.38	0.02	6.02	1.32	27.46
Physician speaker	1.02	−0.37	2.41	2.05	0.15	2.77	0.68	11.12

**Table 5:** Results of UMW test for DISCERN/GQS/JAMA.

	DISCERN	GQS	JAMA
Group name	New	New	New
Group size	26	26	26
Median	52.5	3	2.75
Group name	Old	Old	Old
group size	65	65	65
Median	38.25	2.75	2
*p* value	0.0001	0.02	0.0001

## Data Availability

The original contributions presented in this study are included in the article. Further inquiries can be directed to the corresponding author.
